# Review of Malaysian medicinal plants with potential wound healing activity

**DOI:** 10.1186/s12906-024-04548-5

**Published:** 2024-07-12

**Authors:** Christophe Wiart, Puay Luan Tan, Mogana Rajagopal, Yik-Ling Chew, Mun Yee Leong, Lee Fang Tan, Vi Lien Yap

**Affiliations:** 1 Institute for Tropical Biology & Conservation, Universiti Malaysia Sabah, Kota Kinabalu, Sabah, Malaysia; 2 Faculty of Pharmaceutical Sciences, UCSI University, Cheras, Wilayah Persekutuan Kuala, Lumpur, Malaysia

**Keywords:** *Aloe barbadensis* Miller, *Carica papaya* Linn., *Centella asiatica* Linn., *Cymbopogon nardus* Linn., *Ficus benghalensis* Linn., *Hibiscus rosa sinensis* Linn., Wound healing, Wound repair, Mechanism of action, Angiogenesis

## Abstract

Wound is defined as the damage to biological tissues including skin, mucous membranes and organ tissues. The acute wound heals in less than 4 weeks without complications, while a chronic wound takes longer than 6 weeks to heal. Wound healing occurs in 4 phases, namely, coagulation, inflammatory, proliferative and remodeling phases. Triclosan and benzalkonium chloride are commonly used as skin disinfectants in wound healing. However, they cause allergic contact dermatitis and antibiotic resistance. Medicinal plants are widely studied due to the limited availability of wound healing agents. The present review included six commonly available medicinal plants in Malaysia such as *Aloe barbadensis* Miller, *Carica papaya* Linn., *Centella asiatica* Linn., *Cymbopogon nardus* Linn., *Ficus benghalensis* Linn. and *Hibiscus rosa sinensis* Linn. Various search engines and databases were used to obtain the scientific findings, including Google Scholar, ScienceDirect, PubMed Central and Research Gate. The review discussed the possible mechanism of action of medicinal plants and their active constituents in the wound healing process. In addition, their application in nanotechnology and wound dressings was also discussed in detail.

## Background

Wound is defined as the damage to biological tissues, including skin, mucous membranes and organ tissues [1]. An acute wound heals in less than 4 weeks without complications and is classified into accidental wounds and surgical wounds [2]. Moreover, surgical wounds are classified into 4 classes. Class I (clean wounds) is primarily closed without infection and inflammation. Additionally, Class I wounds do not enter the respiratory, alimentary, genital or urinary tracts. Class II (clean-contaminated wounds) enters the respiratory, alimentary, genital or urinary tracts under controlled conditions. Class III (contaminated wounds) is fresh, open wounds that arise due to the disruption in sterile techniques or the leakage from the gastrointestinal tract into the wound. Class IV (dirty-infected wounds) arises due to improperly managed traumatic wounds and the growth of microorganisms. Thus, devitalized tissues are observed in Class IV wounds [3].

A chronic wound takes longer than 6 weeks to heal. Examples of chronic wounds are diabetic foot ulcers, venous leg ulcers, arterial leg ulcers, and pressure ulcers. The establishment of chronic wounds is attributed to diabetes, vascular disease, ischemia, and neuropathy [2]. According to the National Health and Morbidity Survey 2019, 1 in 5 adults in Malaysia has diabetes, which is approximately 3.9 million Malaysians are diagnosed with diabetes [4]. Hence, there is also an increase in diabetic-related complications such as diabetic foot ulcers (DFU). Hyperglycemia affects the ability of the immune system to eliminate pathogens, while severe infection causes stress hyperglycemia. This two-way mechanism causes diabetic foot infections (DFI), which further delay wound healing and may lead to limb amputation. Empirical antibiotic treatment is crucial due to the unavailability of culture and sensitivity (C&S) results. However, DFU infected with multidrug-resistant organisms (MDROs) makes antibiotic treatment much more challenging [5]. A study conducted at Hospital Kuala Lumpur (HKL) reported that 41.5% of patients with DFU developed DFI. Based on the study, the prevalence of DFI was associated with ethnicity, blood glucose level, blood pressure and cross-sectional area of DFU. Furthermore, patients with a history of amputation, DFI, fasting blood glucose level of ≥ 7 mmol/l, blood pressure of ≥ 140/90 mmHg and DFU size > 10 cm^2^ took a longer period for DFU healing [6].

Triclosan and benzalkonium chloride are commonly used as skin disinfectants in wound healing. However, they cause allergic contact dermatitis. Additionally, cells treated with triclosan may show antibiotic resistance to sulfamethoxazole and ampicillin, while cells treated with benzalkonium chloride may show antibiotic resistance to ampicillin, cefotaxime and sulfamethoxazole [7]. On the other hand, hydrogel-based wound dressings are effective in wound repair due to their ability to keep the wounds hydrated, absorb exudates, relieve pain through cooling effect and adhesion-free properties. However, hydrogels have poor mechanical stability [8]. Hence, medicinal plants are widely studied due to the limited availability of wound healing agents.

A review on the phytochemistry and the mechanisms of wound healing of Vietnamese medicinal plants was published by Hua et al. *Morinda citrifolia* L., *Morus alba* L., *Calophyllum inophyllum* L., *Momordica charantia* L., etc. was shown to promote wound healing [9]. Hosseinkhani et al. reported a review of traditional Persian medicine (*Cocos nucifera* L., *Gentiana lutea* L., *Teucrium polium* L., *Punica granatum* L., *Plantago major* L., etc.) for wound healing [10]. Furthermore, B. Kumar et al. reported the potential wound healing effects of some Indian medicinal plants [11]. However, a review of Malaysian medicinal plants with potential wound healing activity has not been conducted before. Hence, this review mainly focused on six commonly available medicinal plants in Malaysia with potential wound healing properties and their application in nanotechnology and wound dressing.

## Molecular mechanism in wound healing

Generally, wound healing occurs in 4 phases, namely, coagulation, inflammatory, proliferative and remodeling phases. The initial phase of wound healing involves the activation of platelets for clot formation. The whole process is mediated by platelet-derived growth factor (PDGF), transforming growth factor-β (TGF-β), vascular endothelial growth factor (VEGF) and other growth factors or cytokines released by platelets. Cytokines and growth factors act on different target cells and receptors to show their distinctive functions. For instance, PDGF acts as a mitogen and chemotactic agent for fibroblasts and smooth muscle cells that promote angiogenesis and collagen synthesis, in addition to the stimulation of neutrophils and macrophages [12].

The inflammation phase begins 48 h after injury with neutrophils as the predominant cell type. Then the neutrophil count decreases after 24 to 36 h, followed by the migration of monocytes to the wound site. The maturation of monocytes to macrophages plays a role in the inflammatory and repairing phase of wound healing. For instance, macrophages phagocytize wound debris and bacteria and synthesize inflammatory cytokines such as tumor necrosis factor (TNF), interleukin-6 (IL-6), interleukin-1 (IL-1) and basic fibroblast growth factor (bFGF). TNF-α acts as a mitogen for fibroblasts. IL-1 stimulates the proliferation of inflammatory cells and promotes angiogenesis through endothelial cell replication. bFGF acts as a chemotactic and mitogenic factor for fibroblasts, endothelial cells and other mesenchymal cells to regulate angiogenesis. Moreover, bFGF encourages wound contraction, epithelialization and the production of collagen, fibronectin, and proteoglycans. The activation of macrophages (M1 and M2) is dependent on the types of stimuli. Toll-like receptor (TLR)-4 ligands and interferon-γ (IFN-γ) activate M1, thus triggering pro-inflammatory action at the wound site. In contrast, IL-4 and/or IL-13 activate M2 that release macrophage-derived growth factors, PDGF, α fibroblastic growth factor (αFGF), bFGF, transforming growth factor α (TGFα), transforming growth factor β (TGFβ), etc. Hence, the activation of M2 macrophages is vital throughout the wound healing process [12–14]. Autophage also promotes wound repair through antimicrobial, anti-inflammation and immunomodulatory action [15].

During the inflammatory phase, lymphocytes migrate into the wound and release lymphokines, epidermal growth factor (EGF), bFGF, etc. The migration of T lymphocytes is attributed to IL-1 and is crucial for the regulation of collagenase. Optimization of macrophage-derived growth factors and macrophage-derived angiogenic factors at the end of the inflammatory cycle regulates the distribution of fibroblasts, endothelial cells and keratinocytes at the wound site. Furthermore, macrophages manipulate the composition of extracellular matrix (ECM) by stimulating the proliferation of connective, endothelial and epithelial tissue. This is achieved by the release of degrading enzymes and ECM molecules during the angiogenesis and remodeling phase of wound healing [12]. Besides, the inflammatory phase of wound healing involves 5-lipoxygenase pathway that promotes the release of leukotrienes and inducible nitric oxide synthase that generates nitric oxide [16]. Arachidonic acid acts as a precursor to generate leukotrienes and prostaglandins via lipoxygenase pathway and cyclooxygenase pathway, respectively. While the overproduction of leukotrienes and prostaglandins contributes to inflammation due to their chemoattractive effects on eosinophils, neutrophils and macrophages [17].

The proliferation phase begins after the inflammatory phase, whereby fibroblasts start to migrate, proliferate and produce components of the ECM (glycosaminoglycans, proteoglycans and collagen). Collagen is secreted to the extracellular space as procollagen that subsequently cleaves to tropocollagen. Then, tropocollagen cross-links with other tropocollagen to form hydroxylysine and hydroxyproline-rich collagen filaments. The tensile strength of the healing wounds is directly proportional to the extent of cross-link in the collagen. Besides, endothelial progenitor cells (EPCs) regulate angiogenesis and wound revascularization for effective wound healing. The proliferation and migration of both epidermal cells and epithelial cells contribute to re-epithelialization. Cell differentiation such as epithelial-mesenchymal transition (EMT) mediated by β-2 adrenergic receptors (β2-AR) is important due to its ability to establish basement membrane and promote re-epithelialization of wounds. Thus, it plays an important role in accelerating wound healing, while abnormal regulation of EMT causes hypertrophic scar and tissue fibrosis [12]. Lastly, wound contraction takes place during the remodeling phase.

However, the disruption of any one of the wound healing phases results in the development of chronic wound. Chronic wound is also known as non-healing wound, which is manifested by exudation, re-infection, tissue necrosis, defective re-epithelization, decreased angiogenesis and overproduction of reactive oxygen species (ROS). The progression of chronic wound is attributed to persistent inflammatory phase, MMP production, ECM degradation, cell senescence, infections, drug-resistant microbial biofilms, poor vascularization and the unresponsiveness of dermal and/or epidermal cells to reparative stimuli.

The prolongation of inflammatory phase in chronic wounds is due to impaired immune response during the wound healing process. For instance, the amount of pro-inflammatory macrophages increases, while the anti-inflammatory macrophages decline. In addition, the macrophages fail to eliminate dead neutrophils and release matrix metalloproteinases (MMP), i.e., MMP-2 and MMP-9, which degrade ECM and interfere with the proliferation phase of wound healing. MMPs are also secreted by keratinocytes to cause defective in wound re-epithelialization. Impairment of certain micro-ribonucleic acids (miRNAs) such as miR-34a/c, miR-203, miR-19a/b and miR-20a in keratinocytes interrupts immune functions and causes a delay in wound healing. Moreover, the establishment of a highly inflammatory environment is achieved by the accumulation of inflammatory cytokines, i.e., TNF-α and IL-1β. On the other hand, myeloid cells such as macrophages, neutrophils and monocytes persist in the late stage of inflammation. Chemokine receptor CXCR3 is highly expressed in Type 1 T helper (Th1) cells and the ligand for CXCR3 is observed to be over-expressed in chronic inflammation [18, 19].

Infection causes an inflammatory state at the wound bed by activating neutrophils and pro-inflammatory macrophages. As a result, leads to the accumulation of inflammatory cytokines (TNF-α, IL-6, MMP). The accumulation of inflammatory cells produces ROS that cause damaging effects on ECM and cell membranes and eventually leads to premature cell senescence. Moreover, ROS works together with proinflammatory cytokines to stimulate the production of serine proteinases and MMPs that degrade and inactivate ECM components and growth factors which are necessary for normal cell function [18, 19].

Abnormal expression of vascular cell adhesion molecule 1 (VCAM-1) and interstitial cell adhesion molecule 1 (ICAM-1) by endothelial cells promotes cell extravasation that prolongs the inflammatory phase in wound healing. Furthermore, the proliferation phase is delayed due to the lower density of growth factor receptors and the lower mitogenic potential of some growth factors. Although the active cell proliferation marker, Ki-67 is overexpressed in the chronic wound–derived keratinocytes, it shows impairment in migratory potential. Additionally, these cells promote the activation of A-catenin/c-myc pathway and do not express the differentiation markers, especially keratin 10 and keratin 2 [18, 19].

## Experimental models for wound healing

Experimental wound healing models are widely used to study the progression of wound healing. They are divided into in vitro and in vivo models. In vitro wound healing model utilizes cell cultures involving fibroblasts and keratinocytes or a scratch model to investigate for cell viability, proliferation and cytotoxicity [20, 21]. Besides, in vivo model involves laboratory animals with wounds, follows by monitoring of the wound condition over a certain timeframe. Rodents (rats and mice), rabbits and pigs are often used in in vivo models, among these, pig skin is closely related to human skin. However, pigs are prone to infection, costly and difficult to handle and maintain. Hence, the wound healing model is chosen by considering its cost, availability, reproducibility, replicability, ease of handling, similarity to humans and required sample size [20, 22].

Animal models can be subjected to different conditions, namely, incisional wound, excisional wound, burn wound and chronic wound. Incisional wound is created to examine the recovery of surgical wounds, whereas excisional wound is created to investigate wound repair after surgical removal of the skin. Burn wound is created by exposing the skin to hot water (water scalding) or application of direct heat using a hot metal plate. Then, the blister is de-roofed to create an open wound. On the other hand, chronic diabetic wounds can be induced by the administration of alloxan or streptozotocin to promote beta cells destruction in laboratory animals. However, surgical removal of beta cells or the utilization of db/db mice are also commonly practiced. Besides, chorioallantoic membrane (CAM) assay is implemented to study the importance of angiogenesis in wound healing [20, 23].

Excisional wound healing models are vital to study hemorrhage, inflammation, granulation tissue formation, re-epithelialization, angiogenesis and remodeling. In contrast, burn wound healing models are crucial to investigate re-epithelialization, granulation tissue formation, angiogenesis, contraction, scarring and the composition of wound tissues [20].

The wound healing process can be monitored by the following techniques [20]:

1. Wound healing rate (WHR) is calculated using the equation below:

$$Wound\,healing\,rate=\frac{Ai-Af}{Ai}$$

where Ai indicates initial wound area and Af indicates final wound area. The level of re-epithelialization at their respective WHR is shown in Table 1.

**Table 1:** Level of re-epithelialization [20]

Wound healing rate (WHR)	Level of re-epithelialization
< 0	An increase in wound area
0	No re-epithelialization
> 0	A decrease in wound area
1	Complete re-epithelialization

2) Visual presentation of the wound over time using a high-resolution camera captures the morphological changes throughout the wound healing process. The utilization of software such as ImageJ eases the measurement of wound diameter as well as the identification and distribution of various tissues.
3) Biophysical investigation: optical coherence tomography (OCT), confocal laser scanning microscopy (CLSM) and diffuse near-infrared spectroscopy (DNIRS).
4) Histopathological evaluation: hematoxylin & eosin (H&E) to quantify white blood cells in the inflammatory phase and examine blood vessels in angiogenesis. H&E also plays a role in assessing fibroblasts and collagen during the remodeling phase. Additionally, trichromes such as Gomori’s trichrome and Masson’s trichrome stains muscle and intercellular fibers, collagen, cytoplasm, keratin and nuclei.
5) Immunohistochemistry and enzyme-linked immunosorbent assay (ELISA) are utilized to identify and quantify surface markers, cytokines, growth factors, antibodies and antigens.
6) Biochemical assay such as hydroxyproline assay is used to investigate collagen synthesis or metabolism; myeloperoxidase assay is used to study the distribution of neutrophils during the inflammatory phase; N-acetylglucosaminidase assay is used to determine the level of activated macrophages.
7) Flow cytometry is used to analyze the characteristics of cells involved in wound healing.

## Materials and methods

Various search engines and databases, such as Google Scholar, ScienceDirect, PubMed Central and Research Gate, were used to obtain the scientific findings on six commonly available Malaysian medicinal plants with potential wound healing activity. They are *Aloe barbadensis* Miller, *Carica papaya* Linn., *Centella asiatica* Linn., *Cymbopogon nardus* Linn., *Ficus benghalensis* Linn. and *Hibiscus rosa sinensis* Linn. Some of the terms or keywords used to search for potential publications include *Aloe barbadensis* Miller, *Carica papaya* Linn., *Centella asiatica* Linn., *Cymbopogon nardus* Linn., *Ficus benghalensis* Linn., *Hibiscus rosa sinensis* Linn, wound healing, wound repair, wound dressing, chronic wound, etc.

### *Aloe barbadensis* Miller

*Aloe vera* (*Aloe barbadensis* Miller; Family: Asphodelaceae) [24] is well known as lidah buaya in Malay. It has been widely used for the treatment of gingivitis, periodontitis, diabetes, bacterial infections, etc [25]. *Aloe vera* comprises 3 distinctive layers: (1) Inner clear gel that is composed of ≈ 99% water and the remaining glucomannans, amino acids, lipids, sterols, and vitamins. On a dry basis, *aloe vera* gel consists of 55% polysaccharides, 17% sugars, 16% minerals, 7% proteins, 4% lipids, and 1% phenolic compounds [26], (2) The middle layer is made up of latex, which is rich in anthraquinones and glycosides, (3) The outer thick layer provides a protective function and synthesizes carbohydrates and proteins [24].

Razia et al. demonstrated that the combination of processed aloe gel (PAG) and *aloe vera* flower extract (AVF) worked synergistically to promote in vitro wound healing effects. The combination of PAG and AVF (PAG + AVF) increased the cell migration rate and wound healing process after 24 h of treatment in normal human dermal fibroblast (NHDF) cells. However, the wound-healing effect of NHDF cells treated individually with PAG and AVF showed a slower cell migration rate. In addition, PAG + AVF increased the proliferation of NHDF cells by stimulating DNA synthesis. PAG + AVF also enhanced the phosphorylation of protein kinase B (AKT) and extracellular signal-regulated kinase (ERK) as compared to the control and positive control. PAG + AVF promoted angiogenesis by increasing the expression of VEGF and TGF-β. Furthermore, PAG + AVF upregulated the expression of microfibril-associated glycoprotein 4 (MFAP4), type I collagen (COL 1 A), α-smooth muscle (α-SMA), fibrillin and elastin. At the same time, PAG + AVF-treated HaCaT cells showed an increase in cell proliferation and cell migration that promote wound confluence. Anti-inflammatory effects were demonstrated by the decline in inflammatory cytokines such as IL-6 and IL-1β [27].

*Aloe vera* promotes the synthesis of hyaluronic acid and dermatan sulfate for effective wound healing [28]. Hormozi et al. demonstrated that the expression of bFGF and TGF-β1 in mouse embryonic fibroblast cells was upregulated in a dose-dependent and time-dependent manner [29]. TGF-β1 promotes wound healing by recruiting inflammatory cells and inhibiting ECM degradation. Moreover, TGF-β1 stimulates angiogenesis and fibroblast proliferation. Fibroblasts play a crucial role in the production of collagen and fibronectin, which are essential for the formation of ECM [30]. In contrast, bFGF regulates the replication and migration of epithelial, endothelial and fibroblast cells throughout the wound healing process [31].

Nanofibers formulated with ethylcellulose, hydroxypropyl methylcellulose and 10% *aloe vera* (EC/HPMC/Alv) improved cell proliferation and adhesion. This is attributed to the presence of *aloe vera* which increases the hydrophilicity and protein adhesion of nanofibers to NIH-T3T cells. In addition, cells treated with EC/HPMC/Alv did not show cytotoxicity effects regardless of its antibacterial effects against *Staphylococcus aureus* and *Escherichia coli*. EC/HPMC/Alv also showed an increase in tensile strength as compared to nanofibers. Hence, EC/HPMC/Alv is effective for rapid wound healing and as an ideal wound dressing [32].

Polylactic-co-glycolic acid (PLGA)-based nanofiber containing *aloe vera* extract (PLGA-AV) and *aloe vera* extract alone demonstrated antibacterial effects against *Staphylococcus aureus* and *Staphylococcus epidermidis*. PLGA-AV showed an increase in cell proliferation and re-epithelization as compared to control. Furthermore, db/db mice showed a significant improvement in wound closure after 8 days of PLGA-AV treatment [33].

Wound dressing formulated with polyvinyl alcohol, chitosan and *aloe vera* gel (PVA/CS/AV) improved wound closure by 85.93% after 14 days in adult male Wistar rats. However, simultaneous application of PVA/CS/AV and recombinant human erythropoietin (rhEpo) demonstrated a significantly greater wound healing effect (92.96%) than PVA/CS/AV. Furthermore, wounds treated with PVA/CS/AV did not show any signs of wound inflammation or infection [34].

Nanofibers composed of gelatin, *aloe vera* and poly (ε-caprolactone) (Gel/AV-PCL) possessed antibacterial activity against *Staphylococcus aureus* and *Escherichia coli*. These antibacterial effects are attributed to the inhibition of bacterial protein synthesis by anthraquinone-rich *aloe vera*. NIH-3T3 fibroblast cells showed higher cell growth after 4 days of treatment with Gel/AV-PCL. Moreover, a significant increase in cell proliferation was observed [35].

Oral administration of *aloe vera* significantly speeded up recovery of diabetic wounds among Goto-Kakizaki rats. Furthermore, an increase in inflammatory cell infiltration, angiogenesis, epithelialization and ECM deposition was observed. At the same time, the level of TGF-β1 and VEGF was upregulated by *aloe vera* [36].

Acemannan is recognized as one of the major polysaccharides found in *aloe vera* gel. Jettanacheawchankit et al. demonstrated that 2 to 16 mg/mL of acemannan significantly stimulated DNA synthesis and subsequently increased gingival fibroblast proliferation. Furthermore, the expression of keratinocyte growth factor-1 (KGF-1), VEGF and COL 1 A was upregulated by acemannan [37]. Gingival fibroblast plays a vital role in wound healing by generating ECM proteins and growth factors. For instance, KGF-1 accelerates re-epithelialization of the wound, VEGF causes angiogenesis, whereas COL 1 A maintains the structure of ECM for cell adhesion and migration [38–40]. Xing et al. reported that acemannan promoted cell proliferation and wound healing through AKT/mTOR signal pathway and upregulation of cyclin D1 [41]. On the contrary, aloe-derived glucomannan binds specifically to β2-integrins (LFA-01 and Mac-1) in fibroblast. This further enhanced the adhesion, migration and proliferation of fibroblast cells. The growth hormone present in aloe (Gibberellins) also stimulates fibroblast proliferation and protein synthesis [42]. Hence, the wound healing effects of *aloe vera* are mainly contributed to the synergistic effects of acemannan, glucomannan and gibberellins in promoting fibroblast proliferation.

The angiogenic effect of *aloe vera* gel is mainly attributed to β-sitosterol [43]. Aloesin from *aloe vera* is involved in the activation of Smad 2, Smad 3 and mitogen-activated protein kinase (MAPK) signaling pathways. As a result, an increase in the expression of TGF-β1, keratinocyte migration, collagen synthesis and angiogenesis were observed. In vivo study demonstrated that aloesin-treated mice showed a significantly higher wound closure rate than vehicle-treated mice. Furthermore, aloesin-treated mice promoted neoepithelium formation and increased the rate of granulation tissue formation, epidermal regeneration and dermal regeneration [44].

Published studies demonstrated that the administration of topical preparation containing *aloe vera* gel or *aloe vera* extract is generally well tolerated without any adverse effects or serious side effects [45–50]. However, highly concentrated *aloe vera* (98%) is reported to cause erythema and desquamation of skin [51]. Guo and Mei reported that topical and oral administration of *aloe vera* causes skin irritation, urticaria, cramping and diarrhea in patients who are allergic to Liliaceae family. In addition, *aloe vera* gel causes discomfort, pain and hypersensitive reaction [52]. Although *aloe vera* is beneficial in promoting wound healing, its complex chemical nature, concentration, composition and individual contaminants should be taken into consideration during the formulation process. Furthermore, encapsulation of *aloe vera* extract for drug administration provides controlled drug release as compared with direct administration of *aloe vera* extract [49].

### *Carica papaya* Linn

Papaya (*Carica papaya* Linn.; Family: Caricaceae) [53] is widely cultivated in tropical countries such as Malaysia with a self-sufficiency ratio (SSR) of 153.1%. A SSR of more than 100% indicates that the supply of papaya is sufficient to meet local demands [54]. The fruit, juice, seed, root, leaves bark and latex of papaya are rich in phytochemicals, vitamins and minerals. Hence, it is well studied from ancient times till now for its medicinal use. Papaya was proven to have antioxidant, anti-hypertensive, anti-inflammatory, anti-helminthic, antimicrobial, hepatoprotective and wound healing effects [55].

*C. papaya* leaves extract was shown to promote cell proliferation and collagen synthesis throughout the wound healing process. However, its efficacy varies depending on the extraction method and the solvent used in the extraction process [56]. In vivo study demonstrated that *C. papaya* extract significantly reduced the length of the incised oral wound in mice. Hakim et al. also concluded that the topical application of papaya extract promoted epithelization and fibrillation of the wound [57].

Furthermore, ethanolic and aqueous papaya leaf extract showed antibacterial effects against *Coliform bacillus*, *Staphylococcus epidermidis*, *Streptococcus viridans* and *Escherichia coli* [58]. According to Femilian et al., ethanolic papaya leaf extract promoted the healing of buccal ulcers in Wistar rats by regulating macrophage activity, angiogenesis and re-epithelization [59].

Gel formulation containing *C. papaya* L. leaf extract nanoparticles and 1% chitosan increased the number of fibroblasts in the tooth extraction socket of Wistar rats after 7 days. The number of fibroblasts increased proportionally with the concentration of *C. papaya* L. leaf extract. Among the concentrations tested, 25% of the leaf extract of *C. papaya* L. showed a lower number of fibroblasts compared to the positive and negative control [60].

According to Rashid et al., an average of 7 papaya dressings were required to promote the growth of healthy pink granulation tissues among patients with deep burn wounds. This is achieved by the enzymatic proteolytic debridement of deep burn wounds by the topical application of papaya. Therefore, it may help reduce the healthcare burden by preventing the risks associated with surgical debridement. Furthermore, the spilt-thickness skin graft was accepted by the papaya-treated pink granulation tissue bed [61].

A case study in Nigeria utilized unripe pawpaw dressing for the treatment of sacral wounds secondary to chemoradiation. As a result, unripe pawpaw dressing promoted the growth of healthy granulation tissue for wound bed preparation. Furthermore, 100% flap survival and consolidation were observed [62]. Xue et al. demonstrated that the hydrogel containing poly (γ-glutamic acid) (γ-PGA), chitooligo-saccharide, and papain promoted wound healing. Additionally, papain inhibited hypertrophic scar formation by regulating fibroblast proliferation and collagen deposition [63].

Simultaneous use of unripe crushed papaya dressing and leeching was effective in the treatment of nonhealing ulcers. This is attributed to the enzymatic debridement effect of unripe papaya. Enzymatic debridement stimulates the growth of healthy granulation tissue. Moreover, leech saliva is having analgesic, anti-inflammatory, antiplatelet, anticoagulant and antimicrobial effects. Therefore, raw papaya and leeching work synergistically to promote wound healing effects [64]. Unripe papaya pulp acts as an enzymatic wound debriding agent due to the presence of heat-resistant proteolytic enzymes such as papain and chymopapain. It promoted the healing of deep burn wounds by producing healthy granulation tissue and loosened eschar for easy removal. Furthermore, papaya is rich in Vitamin C, which stimulates collagen synthesis [65].

Purified papain preparations were reported to cause severe hypersensitivity reactions but not in papaya fruit. Approximately 1% of papain causes anaphylactic reaction in children [66]. However, papaya fruit dressing should be used cautiously for the first time. Due to the safety issue of purified papain, standardization of papaya extract is necessary for the development of papaya dressings. Furthermore, the identification of other active constituents in papaya and their mechanism for wound healing should be further studied [67]. On the other hand, oral consumption of papaya leaf in the form of juice or standardized aqueous extract is widely used to treat dengue and non-dengue-associated thrombocytopenia. It is generally safe for short term treatment but should be use in caution during pregnancy and in patient with liver impairment. Besides, oral consumption of papaya leaf causes mild gastrointestinal disturbances and rash. Herb-drug interaction was reported for papaya leaf with oral hypoglycaemic drugs such as metformin and glimepiride, p-glycoprotein substrates such as digoxin, antibiotics with cation chelating properties such as ciprofloxacin etc [68, 69]. Thus, a detailed and accurate patient medication history should be taken to avoid coadministration of the drugs with papaya leaf extract. Moreover, in vivo study demonstrated that standardized papaya extract causes minimal fibrosis in spleen [70].

### *Centella asiatica* Linn

*Centella asiatica* Linn. (Family: Apiaceae) [71] is well known as pegaga in Malay. It is widely used in the medical field due to its antimicrobial, anti-inflammatory, anti-cancer, neuroprotective, antioxidant, anti-depressant, antidiabetic, hepatoprotective and wound healing activity [72]. Asiatic acid, madecassic acid, asiaticoside and madecassoside are identified as the active constituents present in *C. asiatica* [73].

Non-cross-linked asiaticoside-polyvinyl alcohol-sodium alginate nanofibers (AT-PVA-SA-NF) showed greater antibacterial activity against *Staphylococcus aureus* and *Pseudomonas aeruginosa* than cross-linked AT-PVA-SA-NF. The cell migration assay demonstrated that nanofibers containing AT promote wound re-epithelialization, which is vital for the wound healing process. The in vivo study demonstrated that the proliferation phase of the nanofiber-treated group started on day 7 with an increased number of fibroblasts and a reduced number of inflammatory cells. In addition, epithelialization and collagen synthesis were observed on day 7 and 14. The study reported that the maturation and remodeling phases of diabetic wound healing were completed on day 14 in the nanofiber-treated group. Therefore, nanofibers containing asiaticoside are effective in healing diabetic wound [74].

The wound healing rate of normal wounds after 14 days of treatment with *Centella asiatica* total glycosides nitric oxide gel (CATGNOG) was dose-dependent (medium dose > low dose > high dose), similar to the diabetic wounds. Whereby, the low dose group contains 4% *C. asiatica* total glycosides, the medium dose group contains 8% *C. asiatica* total glycosides and the high dose group contains 16% *C. asiatica* total glycosides [75]. Nitric oxide promotes wound healing by dilating capillaries, improving microcirculation and regulating growth factors and cytokines [76]. In contrast, *C. asiatica* plays a role in epidermal epithelialization, collagen synthesis and proliferation of fibroblasts [75]. The combination of *C. asiatica* total glycosides and nitric oxide works synergistically to accelerate wound healing in normal and diabetic wounds.

Asiaticoside-treated human foreskin fibroblasts (HFF-1) demonstrated a lower number of inflammatory cells but a greater distribution of fibroblasts, collagen deposition and angiogenesis as compared to the model group. In addition, HFF-1 showed greater cell migration and proliferation which are vital for the repair of diabetic ulcers. In vivo and in vitro studies demonstrated that asiaticoside acts by increasing the expression of Wnt/β-catenin signaling cascade-linked proteins (Wnt1 and Wnt 4), c-myc, total β-catenin and nuclear β-catenin and increasing Glycogen synthase kinase 3β (GSK3β) phosphorylation level [77]. The role of Wnt/β-catenin signaling cascade in wound healing is demonstrated by the regulation of cell migration, proliferation and differentiation [78].

Somboonwong et al. demonstrated that hexane, ethyl acetate, methanol and aqueous extract of *Centella asiatica* increased the healing rate of both incision and burn wounds. Thin-layer chromatography revealed that the main active constituents present in hexane, ethyl acetate and methanol extract were β-sitosterol, asiatic acid and asiaticoside and madecassoside respectively [79]. For instance, β-sitosterol promotes angiogenesis, while asiatic acid induces the expression of TNF Alpha-Induced Protein 6 (TNFAIP6) that regulates ECM synthesis and inflammatory cascades in human fibroblasts [80, 81]. In contrast, asiaticoside upregulates the synthesis of type I collagen via TGFbeta receptor I kinase-independent Smad signaling pathway and exerts antioxidant effects [82]. Additionally, extract-treated groups demonstrated a significant increase in tensile strength, which is attributed to the increase in collagen synthesis. Extract-treated groups also showed complete epithelialization and keratinization without fibrinoid necrosis [79].

Asiaticoside, madecassoside, asiatic acid and madecassic acid were examined for their wound healing effects in vitro and in vivo. RT-PCR showed that asiaticoside and madecassoside significantly increased the mRNA levels of type I and type II collagen, while ELISA demonstrated an increase in the levels of procollagen Type I and Type III. The in vitro study showed that collagen synthesis is mainly attributed to the activation of TGF-β/Smad pathway. This is evidenced by the elevated p-Smad 3 levels, increased TGF-β1 and TβRII mRNA levels and decreased Smad 7 expression. Furthermore, madecassoside demonstrated a greater wound healing effect than asiaticoside. In vivo studies showed that both asiaticoside and madecassoside accelerated wound healing. In contrast, asiatic acid and madecassic acid did not show any wound healing effects in vitro and in vivo [83].

*Escherichia coli*, *Staphylococcus aureus*, *Bacillus subtilis* and *Klebsiella pneumonia* are the most common bacteria that cause wound contamination. Bamboo Fabric Coated *Centella asiatica* (BFC-Ca) was shown to have antibacterial activity against bacteria. Moreover, in vivo study revealed that BFC-Ca-treated albino Wistar rats showed significant wound healing effects within 17 days of treatment as compared to the untreated group. Despite antibacterial effects, BFC-Ca demonstrated excellent air permeability and swelling properties to provide optimal wound healing conditions [84].

The molecular docking of compounds derived from *Centella asiatica* with wound healing associated proteins was examined using Schrödinger Release 2020-2. As a result, madecassic acid, madecassoside and asiatic acid were strongly bound to TNF-α (proinflammatory cytokines). In contrast, asiaticoside showed good binding affinity against Nrf2-Keap1, whereby Nrf2-Keap1 regulated reactive oxygen species (ROS) generation in response to oxidative stress. MM-GBSA reported that madecassic acid inhibited the degradation of antioxidants and collagen; asiatic acid inhibited proinflammatory cytokines, while asiaticoside played a role in collagen synthesis. Hence, the binding affinity of madecassic acid, madecassoside, asiatic acid and asiaticoside to the target sites renders their potential as excellent wound healing agents [85]. Madecassoside, terminoloside and isomadecassoside isolated from *C. asiatica* inhibited lipopolysaccharide-induced nitrite production, thus possessing anti-inflammatory action which may accelerate the wound healing process [86].

Chitosan–aluminum monostearate composite sponge dressing containing asiaticoside was shown to significantly enhance the proliferation of NHDF and normal human epidermal keratinocytes (NHEK). Furthermore, the dressing containing asiaticoside demonstrated dose-dependent angiogenic activity in the chick-chorioallantoic membrane assay. Fibroblast proliferation and angiogenesis work together to promote the healing of chronic wounds [87].

The case report demonstrated that a 15-year-old child and 3 middle-aged women experienced a decline in liver function after taking *C. asiatica*. The consumption of *C. asiatica* is reported to be potentially hepatotoxic due to the presence of pentacyclic triterpene derivatives [88, 89]. Thus, the risk of hepatoxicity should be considered especially if patient is taking other hepatotoxic agents such as tetracycline. High doses of *C. asiatica* may cause headache, mild gastrointestinal disturbances, dizziness and drowsiness [90]. Hence, oral administration of *C. asiatica* should be further studied to determine its efficacy and safety in promoting wound healing. On the other hand, topical administration of *C. asiatica* is generally safe at the recommended dose and may cause allergic reactions and burning [91].

### *Cymbopogon nardus* Linn

Citronella grass (*Cymbopogon nardus* Linn.; Family: Poaceae) [92] is also known as serai wangi in Malay. Hydro-distillation of *C. nardus* yields approximately 3% essential oil. The main compounds identified in essential oil are citral (38.75%), 2,6-octadienal,3,7-dimethyl- (31.02%), citronellal (6.06%), geranyl acetate (4.28%), geraniol (2.75%) and citronellol (1.89%) [93]. *The essential oil of C. nardus* showed antioxidant, anti-inflammatory, antiproliferative, and mosquito repellent activity [92, 94, 95].

The diabetic wound heals slowly and provides favorable conditions for the growth of *Candida* species. The essential oil of *C. nardus* (EO-CN) inhibited the growth of *Candida albicans* (MIC: 25 µg/ml), *Candida glabrata* (MIC: 50 µg/ml) and *Candida tropicalis* (MIC: 50 µg/ml). Furthermore, EO-CN significantly reduced *Candida* load at the infected site. EO-CN renders its anti-inflammatory activity by significantly reducing the levels of inflammatory markers (TNF-α and IL-1β). Both the anti-Candida and anti-inflammatory action of EO-CN accelerated diabetic wound healing [93].

In vivo study demonstrated that citronellal promoted the healing of burn wounds, whereby 1% citronellal showed the fastest recovery rate [96]. Citronella grass extract and its gel preparation showed antibacterial effects against *Staphylococcus aureus* and *Escherichia coli*. However, further tests need to be conducted to identify their MIC and MBC [97]. Antibacterial effects of citronella oil against *Staphylococcus aureus*, *Staphylococcus epidermidis*, *Acinetobacter baumannii*, *Pseudomonas aeruginosa* and *Streptococcus pyogenes* are beneficial to prevent secondary infection of wounds [98]. The antibacterial and antifungal effects of citronella oil are mainly attributed to citronellal [99]. Besides, citronella essential oil and its active compound, geraniol were bactericidal against *Staphylococcus aureus* through biofilm inhibition [100].

Due to the lipophilic nature of essential oils, they bypass the blood-brain barrier and cause neurological toxicity as shown in essential oils of *Salvia officinalis*, *Thuja plicata*, *Eucalyptus* species, etc. The consumption of lavender oil or tea tree oil causes endocrine disruption. Besides, topical application of essential oils causes skin irritation, photosensitization, sensitization and dermatitis [101]. Moreover, the volatility of essential oils leads to the rapid loss of activity, hence, extended-release systems should be utilized to prolong the duration of contact and minimize toxicity effects arising from the use of essential oils.

### *Ficus benghalensis* Linn

Various parts of the Banyan tree (*Ficus benghalensis* Linn.; Family: Moraceae) [102] have their own medicinal benefits. For instance, root possesses anti-glycemic, antibacterial, lipid-lowering and immunostimulatory activity in addition to its role in treating dysentery, pimples, diarrhea and hair fall. Stem bark acts as an anti-arthritic, antibacterial, anti-diabetic, anti-inflammatory, analgesic and antifungal agent. The leaves have hypoglycemic, antibacterial and immunostimulatory action, while latex promotes conception [102, 103]. Compounds such as lanostadienylglucosyl cetoleate, bengalensisteroic acid acetate, protodioscin, leucocyanidin, pelargonidin, lupenyl acetate and lanosterol are present in *Ficus benghalensis* [102].

*F. benghalensis* extract showed greater cell migration at the wound site as compared to silver nitrate and the control group. However, the percentage of wound closure *of F. benghalensis* extract (62%) was lower than silver nanoparticles (70.2%) and rifampicin (84%). Besides, *F. benghalensis* extract showed weaker antibacterial effects than silver nanoparticles and rifampicin. In conclusion, *F. benghalensis* extract accelerated wound healing by promoting cell migration and inhibiting bacterial growth at the wound site [104].

The excision wound healing model was utilized to examine the wound healing effects of *F. benghalensis* leaves which were successively extracted using petroleum ether, 95% ethanol and distilled water. The ointment containing 4% petroleum ether extract showed complete wound healing after 21 days, while the ointment containing 4% ethanolic extract and 4% aqueous extract took 25 days and 28 days respectively, for 100% wound closure. The results were comparable to the positive control (nitrofurazone). Hence, *F. benghalensis* extract is suitable to be used as a wound-healing agent [105].

*F. benghalensis* extract derived from leaves, roots and fruits demonstrated dose-dependent antibacterial effects against *Staphylococcus aureus*, *Escherichia coli*, *Pseudomonas protobacteria* and *Bacillus cereus*. This is vital for the prevention of secondary infection at the wound site due to the maintenance of a bacterial-free environment [106]. Silver nanoparticles formulated with aqueous *F. benghalensis* leaf extract also showed bactericidal activity against *Escherichia coli* [107]. *F. benghalensis* bark extract was shown to have anti-inflammatory action by reducing ROS generation, downregulating the expression of TNF-α and upregulating the expression of IL-10. However, the contribution of its anti-inflammatory action toward wound healing requires further justification [108]. *F. benghalensis* leaves extract showed the greatest wound healing effect in albino Wistar rats as compared to *F. microcarpa*, *F. hispida* and *F. religiosa*. Furthermore, its percentage of wound closure (97.20%) was comparable to Betadine (97.67%) [109].

In vitro wound healing model demonstrated that ethanolic and hydroalcoholic extract of *F. benghalensis* inhibited hemolysis. However, the extract of *F. religiosa* showed greater inhibition of hemolysis than *F. benghalensis* [110]. A high percentage of hemolysis inhibition makes it suitable for topical application due to its excellent blood compatibility [111]. The in vivo wound healing model showed that the ethanolic extract of *F. benghalensis* took 17 days for complete wound healing, whereas the hydroalcoholic extract of *F. benghalensis* took 18 days for complete wound healing, similarly to *F. religiosa* [110].

*In silico* study demonstrated that lupenyl acetate and lanosterol showed anti-inflammatory action through the inhibition of TNF-α and vascular endothelial growth factor receptor (VEGFR), which helps to accelerate diabetic wound healing [112]. Silver nanoparticles synthesized from *F. benghalensis* extract stimulate the production of transforming growth factor-alpha (TGF-α) in a dose-dependent manner [113]. The synthesis of TGF-α promotes endothelial proliferation, migration and angiogenesis which helps to accelerate wound healing [114].

Aqueous extract of *F. benglensis* did not show any signs and symptoms of acute oral toxicity at the dose of 5000 mg/kg in adult albino Wistar strain rats [115]. Khan et al. demonstrated that the LD50 of the aqueous extract of *F. benghalensis* was 2500 mg/kg in random-bred albino rats [116]. To date, there is no adverse effects or death cases reported with the topical use of *F. benghalensis*.

### *Hibiscus rosa sinensis* Linn

Chinese hibiscus (*Hibiscus rosa sinensis* Linn.; Family: Malvaceae) [117] was declared by Malaysia’s first prime minister, Tunku Abdul Rahman as the national flower of Malaysia on 28 July 1960. It is known as Bunga Raya (“celebratory flower”) in Malay. The five petals on the Chinese hibiscus symbolize the five aspects of the National Principles of the Rukun Negara and a symbol of unity [118]. *H. rosa sinensis* is traditionally used as a cough suppressant, hair growth promoter, diuretic and remedies for dysentery, diarrhea, heart diseases, diabetes, epilepsy, leprosy, etc [119].

The ethanolic extract of *Hibiscus rosa sinensis* leaves (EEHRL) showed dose-dependent free radical scavenging activity via DPPH, nitric oxide and superoxide radical scavenging assay. Four different experimental animal models were utilized to study the wound healing effects of EEHRL. The percentage of excision wound closure increased significantly after 15 days of EEHRL treatment. The percentage of excision wound closure and the epithelization period of EEHRL was also comparable to 5% povidone-iodine. In addition, EEHRL increased wound breaking strength in incision wounds, which was comparable to 5% povidone iodine. This may be due to an increase in collagen synthesis and thus stabilization of the collagen fibers. In addition, EEHRL promoted the contraction of burn wounds, most probably by stimulating the growth of keratinocytes and fibroblasts. In dead space wounds, EEHRL-treated wounds showed an increase in granulation tissue weight and hydroxyproline content. Increased hydroxyproline concentration leads to faster collagen turnover and thus accelerates wound healing. EEHRL also showed an increase in antioxidant enzymes such as superoxide dismutase (SOD) and catalase (CAT) in granulation tissue, which helps in the removal of oxidative stress that causes the delay in wound healing [120].

Oral administration of ethanolic *H. rosa sinensis* flower extract demonstrated significantly greater excision wound healing effects as compared to the control group (86% vs. 75%) after 15 days of treatment. Besides, the extract-treated group showed significantly faster epithelialization as compared to the control group (14 days vs. 18 days). Meanwhile, extract-treated incision wounds showed significantly higher tensile strength than the control group. Extract-treated dead space wounds showed a significant increase in granulation tissue weight and hydroxyproline concentration as compared to the control group. Therefore, oral administration of ethanolic *H. rosa sinensis* flower extract possessed an excellent wound healing effect in excision, incision and dead space wound. Granulation tissues of extract-treated wounds consisted of a higher number of collagen and fibroblast, but a lesser number of inflammatory cells as compared to the control group [121].

Incisional wounds of Sprague Dawley rats demonstrated complete re-epithelialization and remodeling of granulation tissues after 15 days of treatment with aqueous and ethanolic extract of *H. rosa sinensis*. Among the extracts, the ethanolic extract showed the highest tensile strength on wounded skin. Tensile strength is mainly attributed to collagen deposition at the wound site. Thus, both extracts are having potential as wound-healing agents due to their in vivo efficacy in incision wounds [122].

N-butyl alcohol extract of *H. rosa sinensis* flowers (NHRS) accelerated excision wound healing by increasing collagen deposition, collagen maturity and fibroblast distribution. Besides, NHRS upregulated the expression of vascular endothelial growth factor (VEGF) and transforming growth factor-β1 (TGF-β1), which are vital for angiogenesis and ECM deposition respectively. The epidermal epithelialization of NHRS-treated wounds was greater than the recombinant bovine basic fibroblast growth factor (rbFGF). The collagen deposition and ECM deposition of extract-treated wounds are attributed to the activation of macrophages and fibroblasts by NHRS. Extract-treated wounds also possessed anti-inflammatory activity by reducing the number of inflammatory cells [123].

The chicken egg chorioallantoic membrane (CAM) assay revealed that the ethanolic extract of *H. rosa sinensis* flowers showed concentration-dependent angiogenesis. Angiogenesis promotes wound healing by replenishing the blood supply to the wound site [124]. The aqueous, ethanol and methanol extract of *H. rosa sinensis* showed antibacterial activity against food poisoning bacteria such as *Staphylococcus aureus*, *Bacillus cereus*, *Clostridium perfringens*, *Listeria monocytogenes*, *Escherichia coli*, *Salmonella typhi* and *Pseudomonas aeruginosa* [125].

Antibacterial agents prevent secondary infection at the wound site, thus promoting wound healing. A nanocomposite made of zinc oxide nanoparticles containing *H. rosa sinensis* leaves extract (ZnO-NPs) and cellulose exhibited antibacterial activity against *Staphylococcus aureus* and *Escherichia coli*. According to Elemike et al., cellulose did not show antibacterial activity, while the nanocomposite showed greater antibacterial acidity against *S. aureus* and *E. coli* as compared to *H. rosa sinensis* leaves extract and ZnO-NPs. This is due to the synergistic effects brought about by the extract and zinc oxide and the larger surface area of the nanocomposite [126]. Silver nanoparticles synthesized using flower, leaf and bark extracts of *H. rosa sinensis* possessed antibacterial activity against *S. aureus*, *B. subtilis*, *P. aeruginosa* and *E. coli*. This is attributed to the large surface area of silver nanoparticles that facilitate the penetration of silver ions into the cell membrane and interrupts the DNA replication of bacteria. Hence, it prevents the multiplication and growth of bacterial cells [127].

The ethanolic extract of *H. rosa sinensis* flowers (EEHRS) showed significantly greater excision wound contraction than the control (untreated) and the reference standard (0.2% nitrofurazone ointment). Furthermore, complete healing was observed at 18 days and 16 days for 5% EEHRS ointment and 10% EEHRS ointment respectively. The incision wound model demonstrated a significant increase in tensile strength after treatment with 5% and 10% EEHRS ointment as compared to the control group. The increase in tensile strength is attributed to the stimulation of collagen synthesis and maturation by EEHRS. On the other hand, the dead space wound model treated with 5% and 10% EEHRS ointment showed a significant increase in wet granuloma weight, dry granuloma weight and tensile strength as compared to the control group. The biochemical evaluation of wet granulation tissues showed a significantly higher DNA, protein, collagen, hexosamine and uronic acid content on days 4, 8 and 12, as compared to the control group. Among these, hexosamine and uronic acid play a role in the synthesis of extracellular matrix (ECM) [128].

Swiss albino mice treated with green nanofiber mat synthesized from hibiscus leaves mucilage-Pectin-Polyvinyl alcohol (HLM-Pectin-PVA) were shown to have greater collagen and fibroblast growth as compared to the control group. Furthermore, HLM-Pectin-PVA demonstrated faster wound healing (85.71% vs. 60%) and wound contraction (30 mm^2^ vs. 21 mm^2^) than the control group after 8 days of treatment. The non-hemolytic nature of HLM-Pectin-PVA makes it suitable as a wound dressing due to its hemocompatibility [129]. An herbal ointment containing *H. rosa sinensis* flower extract and *Curcuma longa* rhizomes extract works synergistically to speed up the wound healing process. This is attributed to the anti-inflammatory and antioxidant effects of the active constituents present in the extracts [130].

Acute oral toxicity study in Swiss albino mice demonstrated that the methanolic extract of *H. rosa sinensis* leaf was relatively safe with LD50 of more than 2000 mg/kg, which is similar to hydroalcoholic extract of *Hibiscus rosa sinensis* flower in Wistar rats. Kandhare et al. demonstrated that hydroalcoholic extract of *H. rosa sinensis* leaf was safe up to 5000 mg/kg in Swiss albino mice. Subacute toxicity study of the extract did not show any adverse effects at 400 mg/kg, but hepatotoxicity and renal toxicity were observed at 800 mg/kg of extract [131–133]. Besides, crude *H. rosa sinensis* flower extract demonstrated anti-fertility effect in male albino rats, which is beneficial for birth control [134]. In vivo study demonstrated that the oral administration of aqueous *H. rosa sinensis* flower extract increased cardiovascular risk in non-diabetic pregnant Wistar rats. In addition, the extract possessed anti-implantation, abortion and anti-fertility effects which may be harmful to healthy, pregnant women. Hence, *H. rosa sinensis* flower extract should be avoided during pregnancy [135]. To date, there is no adverse effects or death cases reported with the topical use of *H. rosa sinensis*.

It can be concluded that wound healing is accelerated by the increase in the rate of cell proliferation, cell migration, angiogenesis, epithelialization, extracellular matrix deposition and collagen synthesis. In addition, the antibacterial, antioxidant and anti-inflammatory actions of Malaysian medicinal plants provide an additional advantage for the recovery of wounds. The wound healing effects may be attributed to the whole medicinal plant extract or the specific compound present in the medicinal plant or the synergistic effects of the medicinal plant/compound to other herbs/compounds. Thus, the study of possible interaction between one medicinal plant towards others is crucial to ensure that the combination is optimal for wound healing. Besides, the interaction between the medicinal plant extract, excipients (e.g.: polymer/dressing) and packaging materials (e.g.: glass/plastic) should also be considered to ensure the stability, quality and safety of the final product. Table 2 summarizes six Malaysian medicinal plants with potential wound healing activity. Currently, wound healing preparations containing *Cymbopogon nardus* Linn., *Ficus benghalensis* Linn. and *Hibiscus rosa sinensis* Linn. are not available in the market. However, wound healing preparations containing *Aloe barbadensis* Miller, *Carica papaya* Linn. and *Centella asiatica* Linn. are available in the market as shown in Table 3.

**Table 2:** Malaysian medicinal plants with potential wound healing activity

Medicinal plant	Active	Study	Model/method used	Mechanism of action	Reference
	ingredient				
***Aloe barbadensis*** **Miller**	50 µg/mL processed aloe gel + 25 µg/mL or 100 µg/mL *aloe vera* flower extract	*In vitro*	Cell migration assay, BrdU cell proliferation assay, scratch wound healing assay, qRT-PCR, western blotting, ELISA, MFAP 4 specific siRNA knockdown	Increases cell migration rate	[27]
				Increases proliferation of NHDF cells	
				Increases the expression of VEGF and TGF-β	
				Reduces inflammatory cytokines (IL-6 and IL-1β)	
				Upregulates the expression of MFAP4, COL 1A, α-SMA, fibrillin and elastin	
	Nanofibers containing 3% *aloe vera*	*In vitro*	MTS assay	Promotes the synthesis of hyaluronic acid and dermatan sulfate	[28]
	50, 100 and 150 µg/mL *aloe vera* gel	*In vitro*	Mouse embryonic fibroblasts, RT-PCR, ELISA	Increases the expression of bFGF and TGF-β1	[29]
	Nanofibers containing 10% *aloe vera* powder	*In vitro*	NIH-3T3 cell viability, SEM technique	Increases cell proliferation and adhesion	[32]
			Agar disc diffusion assay	Antibacterial effects against *S. aureus* and *E. coli*	
	Nanofibers containing *aloe vera* extract	*In vitro*	Agar disc diffusion assay	Antibacterial effects against *S. aureus* and *S. epidermidis*	[33]
			BalbC/3T3 A31 fibroblast bioactivity assay	Increase in cell proliferation	
		*In vivo*	Full-thickness wound healing assay in male db/db mice	Significant improvement in wound closure and reepithelization	
	Wound dressing containing *aloe vera* gel	*In vivo*	Full-thickness excisional wound model in male Wistar rats	Improves the wound closure	[34]
				Anti-inflammation	
	Nanofibers containing 5% or 10% *aloe vera* powder	*In vitro*	Disc diffusion, broth dilution method	Antibacterial activity against *S. aureus* and *E. coli*	[35]
			Cell-scaffold interaction, FE-SEM	Increases the cell growth of NIH-3T3 fibroblast cells	
			Colorimetric MTT assay	Increases cell proliferation	
	30 mg lyophilized *aloe vera* powder dissolved in 1.5 mL purified water	*In vivo*	Full-thickness skin wound excision in Goto-Kakizaki rats	Speeds up the recovery of diabetic wounds	[36]
				Increases inflammatory cell infiltration, angiogenesis, epithelialization and extracellular matrix deposition	
				Increases TGF-β1 and VEGF	
	Acemannan	*In vitro*	Human gingival fibroblasts, [3H]-thymidine incorporation assay	Stimulates DNA synthesis	[37]
				Increases gingival fibroblast proliferation	
			ELISA	Increase the expression of KGF-1, VEGF and COL 1A	
	Carbopol® containing 0.5%, 1% and 2% acemannan	*In vivo*	Punch biopsy wounds from the hard palate of male Sprague Dawley rats	Reduces oral wound areas	
				Increase re-epithelialization	
	Acemannan	*In vitro*, *in vivo*	^35^S-methionine incorporation assay, phosphorylation of AKT in acemannan-stimulated fibroblast cells, m^7^ GTP pull-down assay, full-thickness skin excisional wound model in male BALB/c mice, *in vivo* block of mTOR signaling by rapamycin	Promotes wound healing through AKT/mTOR signal pathway	[41]
				Upregulates cyclin D1	
	Glucomannan	*In vitro*	FE-SEM	Binds to β2-integrins (LFA-01 and Mac-1) on fibroblast	[42]
	Gibberellins	*In vitro*	MTS assay	Stimulates fibroblast proliferation and protein synthesis	
	β-sitosterol	*In vitro*	Chorioallantoic membrane assay	Enhances angiogenesis	[43]
	Aloesin	*In vitro*	Western blotting, ELISA	Activation of the Smad 2, Smad 3 and MAPK signaling pathways. Increases the expression of TGF-β1	[44]
			Scratch wound healing assay	Promotes keratinocyte migration	
			Masson’s trichrome staining	Promotes collagen synthesis	
			Endothelial cell tube formation assay	Promotes angiogenesis	
	0.1% and 0.5% aloesin solution	*In vivo*	Full-thickness wounds in SKH-1 hairless mice	Promotes the formation of neoepithelium	
				Increases the rate of granulation tissue formation, epidermal regeneration and dermal regeneration	
***Carica papaya*** **Linn.**	Methanolic leaf extract	*In vitro*	Scratch assay	Promotes cell proliferation	[56]
			Sircol collagen assay	Promotes collagen synthesis	
	25%, 50% and 75% fruit extract	*In vivo*	Incised wound in *Mus musculus*	Promotes epithelization and fibrillation of the wound	[57]
				Reduces the length of the incised oral wound in mice	
	Ethanolic aqueous leaf extract	*In vitro*	Disc diffusion, broth dilution method	Antibacterial effects against *C. bacillus*, *S. epidermidis*, *S. viridans* and *E. coli*	[58]
	Ethanolic leaf extract	*In vivo*	Oral ulcers in Wistar rats	Regulates the activity of macrophages, angiogenesis and re-epithelization	[59]
	Papaya paste	Clinical study	Deep burn wound	Promote the growth of healthy granulation tissue	[61]
				Enzymatic proteolytic debridement	
	Unripe pawpaw dressing	Case report	Sacral radiation ulcer	Promotes the growth of healthy granulation tissue	[62]
	Hydrogel containing 0.4%, 1%, 2% and 4% papain	*In vitro*	NIH/3T3 fibroblasts activity	Regulates fibroblasts proliferation	[63]
	Hydrogel containing 4% papain	*In vivo*	H&E staining	Inhibits the formation of hypertrophic scar	
			Masson staining	Regulates collagen deposition	
	Unripe crushed papaya dressing	Case study	Non-healing chronic ulcer	Enzymatic debridement effect	[64]
	Papain, chymopapain, vitamin C	Clinical study	Second-degree and third-degree burns	Promotes the growth of healthy granulation tissue	[65]
				Loosens eschar for easy removal	
				Stimulates collagen synthesis	
***Centella asiatica*** **Linn.**	Nanofibers containing asiaticoside	*In vitro*	Agar well disc diffusion method, time kill assay	Antibacterial activity against *S. aureus* and *P. aeruginosa*	[74]
			*In vitro* scratch assay	Promotes wound re-epithelialization	
		*In vivo*	Single circular full thickness wound in albino Wistar male rats	Increases fibroblast proliferation	
				Reduces inflammatory cells	
				Stimulates epithelialization and collagen synthesis	
	Gel containing 8% *C*. *asiatica* total glycosides	*In vivo*	Diabetic cutaneous ulcers in Sprague Dawley rats	Promotes epidermal epithelialization	[75]
				Promotes collagen synthesis	
				Stimulates fibroblast proliferation	
	Fabric coated *C. asiatica* extract	*In vitro*	Fabric diffusion method	Antibacterial activity against *E. coli*, *S. aureus*, *B. subtilis* and *K. pneumonia*	[84]
		*In vivo*	Wound on dorsal side of male albino Wistar rats	Significant wound healing effects	
	Dressing containing 0.012% asiaticoside	*In vitro*	Cytotoxicity study	Enhance the proliferation of NHDF and NHEK	[87]
			Chick-chorioallantoic membrane assay	Promotes angiogenesis	
	Asiaticoside	*In vitro*, *in vivo*	H&E staining	Reduces the number of inflammatory cells	[77]
				Increases fibroblast distribution	
				Enhance angiogenesis	
			Masson staining	Increases collagen deposition	
			CCK8 assay, transwell assay, flow cytometry	Stimulates cell migration and proliferation	
			Diabetic rats with full-thickness wounds, western blotting	Increases the expression of Wnt/β-catenin signaling cascade-linked proteins (Wnt1 and Wnt 4), c-myc, total β-catenin and nuclear β-catenin	
				Increases GSK3β phosphorylation level	
	10% hexane, ethyl acetate, methanol and aqueous extract	*In vivo*	Male Sprague Dawley rats with incision and burn wound	Increases the healing rate of incision and burn wounds	[79]
				Increases in tensile strength by collagen synthesis	
				Complete epithelialization and keratinization	
	β-sitosterol	*In vitro*	Chick embryo chorioallantoic membrane assay	Promotes angiogenesis	[81]
	Asiatic acid	*In vitro*	cDNA microarrays, RT-PCR	Induces the expression of TNFAIP6	[80]
	Asiaticoside	*In vitro*	Smad immunoprecipitation and blotting, western blotting	Upregulates the synthesis of COL 1A via TGF-β receptor I kinase-independent Smad signaling pathway	[82]
	Asiaticoside	*In vitro*, *in vivo*	RT-PCR, ELISA, western blotting, full-thickness burn injury in male ICR mice	Increases the mRNA levels of type I and type II collagen	[83]
				Increase in procollagen type I and type III levels	
				Activation of TGF-β/Smad pathway	
				Accelerates wound healing	
	Madecassoside	*In vitro*, *in vivo*	RT-PCR, ELISA, western blotting, full-thickness burn injury in male ICR mice	Increases the mRNA levels of type I and type II collagen	
				Increase in procollagen type I and type III levels	
				Activation of TGF-β/Smad pathway	
				Accelerates wound healing	
	Madecassic acid	*In silico*	Glide module, grid-based docking protocol, glide extra precision (XP) docking protocol, Qik-Prop, Prime/MM-GBSA module	Strongly bound to TNF-α	[85]
				Inhibits the degradation of antioxidants and collagen	
	Madecassoside	*In silico*	Glide module, grid-based docking protocol, glide extra precision (XP) docking protocol, Qik-Prop, Prime/MM-GBSA module	Strongly bound to TNF-α	
	Asiatic acid	*In silico*	Glide module, grid-based docking protocol, glide extra precision (XP) docking protocol, Qik-Prop, Prime/MM-GBSA module	Strongly bound to TNF-α	
				Inhibits proinflammatory cytokines	
	Asiaticoside	*In silico*	Glide module, grid-based docking protocol, glide extra precision (XP) docking protocol, Qik-Prop, Prime/MM-GBSA module	Good binding affinity against Nrf2-Keap1	
				Promotes collagen synthesis	
	Madecassoside, terminoloside, isomadecassoside	*In vitro*	Colorimetric nitrite assay	Inhibits lipopolysaccharide-induced nitrite production	[86]
***Cymbopogon nardus*** **Linn.**	Ethanolic extract of leaves and stem	*In vitro*	Disc diffusion method, macro dilution test	Antibacterial effects against *S. aureus* and *E. coli*	[97]
	Antiseptic gel containing 1% citronella grass extract	*In vitro*	Disc diffusion method, macro dilution test	Antibacterial effects against *S. aureus* and *E. coli*	
	Essential oil	*In vitro*	Agar well diffusion method, broth macro dilution method	Inhibits the growth of *C. albicans*, *C. glabrata* and *C. tropicalis*	[93]
	25 µg essential oil in 100 mL olive oil	*In vivo*	Fungal infected diabetic wound in Swiss albino mice	Effectively eradicates *C. albicans* colonization and accelerates wound closure	
				Reduces the levels of inflammatory markers (TNF-α and IL-1β)	
	Essential oil	*In vitro*	Broth microdilution method	Antibacterial effects against *S. aureus*, *S. epidermidis*, *A. baumannii*, *P. aeruginosa* and *S. pyogenes*	[98]
	Essential oil	*In vitro*	Disc diffusion method, broth microdilution method	Bactericidal against *S. aureus*	[100]
	Geraniol	*In vitro*	Disc diffusion method, broth microdilution method	Bactericidal against *S. aureus*	
	0.25%, 0.5% and 1% citronellal ointment	*In vivo*	Burn wound in *Mus musculus*	Promotes the healing of burn wounds	[96]
	Citronellal	*In vitro*	Disc diffusion method, serial dilution method	Antibacterial and antifungal effects	[99]
***Ficus benghalensis*** **Linn.**	Aqueous bark extract	*In vitro*	Cell scratch assay	Promotes cell migration at wound site	[104]
			Kirby-Bauer disk diffusion method	Antibacterial effects	
	Ointment containing 4% petroleum ether, ethanolic and aqueous leaf extract	*In vivo*	Excision wound healing model in male adult albino rats	Promotes wound healing and wound closure	[105]
	Ethanolic extract	*In vitro*	Disc diffusion method	Antibacterial effects against *S. aureus*, *E. coli*, *P. protobacteria* and *B. cereus*	[106]
	Methanolic bark extract	*In vitro*	Nitroblue tetrazolium assay, nitric oxide assay, lipid peroxidation assay	Anti-inflammatory action	[108]
			PCR	Downregulates the expression of TNF-α expression and upregulates the expression of IL-10	
	10% aqueous leaf extract ointment	*In vivo*	Excision wound model in albino Wistar rats	Greatest wound healing effects among the *Ficus* species	[109]
				Comparable percentage of wound closure with Betadine	
	Ethanolic and hydroalcoholic extract	*In vitro*	Red blood cells membrane stabilization	Inhibits hemolysis	[110]
	Gel containing 10% ethanolic/ hydroalcoholic extract	*In vivo*	Excision wound model in rats	Complete wound healing after 17–18 days	
	Nanoparticles synthesized from extract	*In vitro*	ELISA	Stimulates the production of TGF-α	[113]
	Nanoparticles containing aqueous leaf extract	*In vitro*	Microdilution method	Bactericidal activity against *E. coli*	[107]
	Lupenyl acetate, lanosterol	*In silico*	Discovery Studio software	Inhibits TNF-α and VEGFR	[112]
***Hibiscus rosa sinensis*** **Linn.**	Ethanolic leaves extract	*In vitro*	DPPH, nitric oxide, superoxide radical scavenging activity	Free radical scavenging activity	[120]
	10% ethanolic leaves extract ointment	*In vivo*	Excision wound model in Swiss albino mice	Significantly increase the percentage of excision wound closure	
			Incision wound model in Swiss albino mice	Increases wound breaking strength in incision wounds by increasing collagen synthesis	
			Burn wound model in Swiss albino mice	Stimulates the growth of keratinocytes and fibroblasts	
	200 mg/kg ethanolic leaves extract		Dead space wound model in Swiss albino mice	Increases granulation tissue weight and hydroxyproline content of dead space wound	
			*In vivo* antioxidant assay	Increase in antioxidant enzymes (SOD, CAT) in granulation tissue	
	120 mg/kg ethanolic flower extract	*In vivo*	Masson’s trichrome staining	Stimulates the synthesis of collagen and fibroblast	[121]
				Reduces the number of inflammatory cells in granulation tissue	
			Excision wound model in Sprague Dawley rats	Significantly enhance excision wound healing effects	
				Increases the rate of epithelialization in excision wound	
			Incision wound model in Sprague Dawley rats	Increases the tensile strength in incision wound	
			Dead space wound model in Sprague Dawley rats	Significantly increase the granulation tissue weight and hydroxyproline concentration in dead space wounds	
	Aqueous and ethanolic extract	*In vivo*	Incision wound model in Sprague Dawley rats	Promotes re-epithelialization and remodeling of granulation tissues in incision wounds	[122]
				Increases the tensile strength in wounded skin	
	80, 160 and 320 mg/mL N-butyl alcohol flower extract	*In vivo*	Excision wound model in Sprague Dawley rats, H&E staining, Masson’s trichrome staining, immunohistochemistry	Increases the collagen deposition, collagen maturity and fibroblast distribution	[123]
				Promotes epidermal epithelialization of wounds	
				Upregulates the expression of VEGF and TGF-β1	
				Anti-inflammatory effects	
	Ethanolic flower extract	*In vivo*	Chicken egg chorioallantoic membrane assay	Promotes angiogenesis	[124]
	Aqueous, ethanol and methanol extract	*In vitro*	Agar diffusion assay, micro-dilution method	Antibacterial activity against *S. aureus*, *B. cereus*, *C. perfringens*, *L. monocytogenes*, *E. coli*, *S. typhi* and *P. aeruginosa*	[125]
	Nanocomposite containing leaves extract	*In vitro*	Agar well diffusion method	Antibacterial effects against *S. aureus* and *E. coli*	[126]
	Silver nanoparticles synthesized using flowers, leaves and bark extract	*In vitro*	Not specified	Antibacterial activity against *S. aureus*, *B. subtilis*, *P. aeruginosa* and *E. coli*	[127]
	Ointment containing 5% and 10% ethanolic flower extract	*In vivo*	Excision wound model in Wistar albino rats	Significantly improves excision wound contraction	[128]
				Complete wound healing at 18 days (5% ointment) and 16 days (10% ointment)	
			Incision wound model in Wistar albino rats	Increase in tensile strength of incision wounds	
			Dead space wound model in Wistar albino rats	Significantly increases wet granuloma weight, dry granuloma weight and tensile strength of dead space wound	
				Increases DNA, protein, collagen, hexosamine and uronic acid content of wet granulation tissue	
	Nanofibers synthesized from 1% leaves mucilage	*In vivo*	Incision wound model in Swiss albino mice	Promotes collagen and fibroblast growth	[129]
			Excision wound model in Swiss albino mice	Faster wound healing and wound contraction	
			Hemo-compatibility analysis	Non-hemolytic	
	Ointment containing ethanolic flower extract	*In vivo*	Excision wound model in Sprague Dawley rats	Anti-inflammatory and antioxidant effects	[130]

**Table 3:** Wound healing preparations available in the market

Product name	Ingredient	Manufacturer	Reference
Lucas’ Papaw Ointment	Fermented *C. papaya* fresh fruit, 39 mg/g	Lucas’ Papaw Remedies©	[136]
Real Paw Paw Ointment	Fermented pawpaw, 80 mg/g	Real Paw Paw	[137]
Théra Wise Natural Wound Care Ointment	Aloe extract	Derma Wise Skin Care Ltd.	[138]
Beaphar Wound Ointment	*Aloe vera*	Beaphar UK Ltd.	[139]
I’m Wounded! Natural Wound Cream	*Aloe vera*	Holistic Healer & Wellness	[140]
Elements Wellness Wound Healing Cream	*Aloe vera*	Elements Wellness Pvt. Ltd.	[141]
WoundKreme™ Organic Healing Ointment	*Aloe vera*	Feuilleorganix Sdn. Bhd.	[142]
Melcura™ HoneyPlus Honey-Based Wound Ointment	*Aloe vera*	Medika SA Trading	[143]
GoHeal Antiseptic Skin Ointment	*Aloe vera*	Naturaline Nutraceuticals Pvt. Ltd.	[144]
Hurix’s Aloe Vera Plus Cream	*Aloe vera*	J.B. Pharmacy Group Sdn. Bhd.	[145]
Scar and Wound Healing Cream	*Aloe vera*	Natural Scents Aromatherapy	[146]
Nemoderm® Wound Healing Cream	*Aloe vera* juice	FAME Pharmaceuticals Industry Co., Ltd.	[147]
Centella Cream	*C. asiatica*	Abhaibhubejhr Thai Herbal Medicine Museum	[148]
Madecare Ointment	*C. asiatica* quantitative extract, 1 g (400 mg as asiaticoside)	Firson Co., Ltd	[149]
FUYUAN *Centella asiatica* Cream Ointment	*C. asiatica, 2.5%*	Hainan Poly Pharm. Co., Ltd.	[150]

## Limitations and strengths

The present review focused on only six commonly available Malaysian medicinal plants with potential wound healing properties. However, many medicinal plants were not included in this review or are still in the discovery phase. Moreover, the source, method of cultivation, processing, extraction and storage of the medicinal plants are not standardized. This led to the variation in phytochemicals content which may affect the efficacy and safety of the end products. Hence, the standardization of medicinal plant extract is crucial to provide quality assurance. Additionally, the active constituents possessing wound healing properties in some medicinal plants have not yet been identified. Clinical trials should be conducted on medicinal plants to demonstrate their clinical efficacy and long-term safety after administration. Furthermore, the maximum and minimum concentrations of medicinal plant extract to be used should be identified to avoid treatment failure and toxicity. Researchers are encouraged to disclose the concentration of extracts or compounds upon publication of the original research paper to ensure the reproducibility and replicability of the experiment. In this review, some of the cellular and molecular aspects of medicinal plants in wound healing are briefly discussed. More research should be conducted in the future to reveal their exact biologically active cellular and molecular mechanisms. Thus, this review acts as a stepping stone to discover the wound healing mechanism of medicinal plants with potential wound healing activity in detail. *In silico* modeling should be utilized in the future to discover the pharmacologic effects or mechanisms of active constituents or herbal extracts in the wound healing process. The research on the pharmacokinetics and bioavailability of medicinal plants and their respective compounds is still lacking, thus further research should be conducted to determine the interaction of the body with the medicinal plants and their respective compounds. Pharmacokinetics and bioavailability are crucial to ensure that appropriate amounts of medicinal plants or active constituents are delivered to the target sites for wound healing effects and to minimize toxicity at the same time. The present review is the first review of Malaysian medicinal plants with wound healing properties. The present review also included the in vitro and in vivo wound healing effects of the plant extracts, the active constituents and the formulations containing plant extract.

## Conclusions

The identification of medicinal plants with wound healing potential is crucial due to the limited availability of wound healing agents and the risk of allergic contact dermatitis and antibiotic resistance with the use of current skin disinfectants such as triclosan and benzalkonium chloride. This review summarizes that *Aloe barbadensis* Miller, *Carica papaya* Linn., *Centella asiatica* Linn., *Cymbopogon nardus* Linn., *Ficus benghalensis* Linn. and *Hibiscus rosa sinensis* Linn. demonstrated wound healing properties through various in vitro and in vivo studies. However, clinical trials should also be conducted to ensure the efficacy, quality and safety of medicinal plants as wound healing agents. Moreover, further research is required to assess the short-term and long-term safety/toxicity of medicinal plants. The safest route of administration of the medicinal plant extract for wound healing should be selected by considering their ease of administration, patient compliance, pharmacokinetics, toxicity and use in pregnancy.

## Data Availability

All data generated or analyzed during this study are included in this published article.

## Abbreviations

α-SMA: α-smooth muscle
bFGF: Basic fibroblast growth factor
CAM: Chorioallantoic membrane
CCK8: Cell Counting Kit8
CLSM: Confocal laser scanning microscopy
COL 1A: Type I collagen
DPPH: 1,1-diphenyl-2- picrylhydrazyl
DNIRS: Diffuse near-infrared spectroscopy
ELISA: Enzyme-linked immunosorbent assay
FE-SEM: Field emission scanning electron microscopy
GSK3β: Glycogen synthase kinase 3β
H&E: Hematoxylin and eosin
IL-1β: Interleukin-1β
IL-6: Interleukin-6
IL-10: Interleukin-10
KGF-1: Keratinocyte growth factor-1
LFA-01: Lymphocyte function-associated antigen 1
Mac-1: Macrophage-1 antigen
MAPK: Mitogen-activated protein kinase
MFAP4: Microfibril-associated glycoprotein 4
MTS assay: (3-(4,5-dimethylthiazol-2-yl)-5-(3-carboxymethoxyphenyl)-2-(4-sulfophenyl)-2 H-tetrazolium) assay
MTT: 3-(4,5-dimethylthiazol-2-yl)-2,5-diphenyl-2 H-tetrazolium bromide
NHDF: Normal human dermal fibroblast
NHEK: Normal human epidermal keratinocyte
NIH-3T3: Mouse embryonic fibroblast cell line
OCT: Optical coherence tomography
qRT-PCR: Quantitative real time-PCR
RT-PCR: Reverse transcription polymerase chain reaction
SEM: Scanning electron microscope
siRNA: Small interfering RNA
TNFAIP6: TNF Alpha Induced Protein 6
TGF-α: Transforming growth factor-α
TGF-β: Transforming growth factor-β
TGF-β1: Transforming growth factor-β1
TNF-α: Tumor necrosis factor-α
VEGF: Vascular endothelial growth factor
VEGFR: Vascular endothelial growth factor receptor
WHR: Wound healing rate
A. baumannii: Acinetobacter baumannii
B. cereus: Bacillus cereus
B. subtilis: Bacillus subtilis
C. albicans: Candida albicans
C. glabrata: Candida glabrata
C. tropicalis: Candida tropicalis
C. perfringens: Clostridium perfringens
C. bacillus: Coliform bacillus
E. coli: Escherichia coli
K. pneumonia: Klebsiella pneumonia
L. monocytogenes: Listeria monocytogenes
P. aeruginosa: Pseudomonas aeruginosa
P. protobacteria: Pseudomonas protobacteria
S. aureus: Staphylococcus aureus
S. epidermidis: Staphylococcus epidermidis
S. pyogenes: Streptococcus pyogenes
S. typhi: Salmonella typhi
S. viridans: Streptococcus viridans
